# Rhinitis, Asthma and Respiratory Infections among Adults in Relation to the Home Environment in Multi-Family Buildings in Sweden

**DOI:** 10.1371/journal.pone.0105125

**Published:** 2014-08-19

**Authors:** Juan Wang, Karin Engvall, Greta Smedje, Dan Norbäck

**Affiliations:** Department of Medical Sciences, Uppsala University and University Hospital, Uppsala, Sweden; Kliniken der Stadt Köln gGmbH, Germany

## Abstract

Risk factors for rhinitis, asthma and respiratory infections in the home environment were studied by a questionnaire survey. Totally 5775 occupants (≥18 years old) from a stratified random sample of multi-family buildings in Sweden participated (46%). 51.0% had rhinitis in the last 3 months (current rhinitis); 11.5% doctor diagnosed asthma; 46.4% respiratory infections in the last 3 months and 11.9% antibiotic medication for respiratory infections in the last 12 months. Associations between home environment and health were analyzed by multiple logistic regression, controlling for gender, age and smoking and mutual adjustment. Buildings constructed during 1960–1975 were risk factors for day time breathlessness (OR = 1.53, 95%CI 1.03–2.29). And those constructed during 1976–1985 had more current rhinitis (OR = 1.43, 95%CI 1.12–1.84) and respiratory infections (OR = 1.46, 95%CI 1.21–1.78). Cities with higher population density had more current rhinitis (*p* = 0.008) and respiratory infections (*p*<0.001). Rented apartments had more current rhinitis (OR = 1.23, 95%CI 1.07–1.40), wheeze (OR = 1.20, 95%CI 1.02–1.41), day time breathlessness (OR = 1.31, 95%CI 1.04–1.66) and respiratory infections (OR = 1.13, 95%CI 1.01–1.26). Living in colder parts of the country was a risk factor for wheeze (*p* = 0.03) and night time breathlessness (*p* = 0.002). Building dampness was a risk factor for wheeze (OR = 1.42, 95%CI 1.08–1.86) and day time breathlessness (OR = 1.57, 95%CI 1.09–2.27). Building dampness was a risk factor for health among those below 66 years old. Odor at home was a risk factor for doctor diagnosed asthma (OR = 1.49, 95%CI 1.08–2.06) and current asthma (OR = 1.52, 95%CI 1.03–2.24). Environmental tobacco smoke (ETS) was a risk factor for current asthma (OR = 1.53, 95%CI 1.09–2.16). Window pane condensation was a risk factor for antibiotic medication for respiratory infections (OR = 1.41, 95%CI 1.10–1.82). In conclusion, rhinitis, asthma and respiratory infections were related to a number of factors in the home environment. Certain building years (1961–1985), building dampness, window pane condensation and odor in the dwelling may be risk factors.

## Introduction

Concern about possible health effects of indoor air pollution has been increasing especially with respects to allergies and asthma. The dwelling is an important indoor environment, since it is where we spend most time, usually 15–16 h per day [1].

Rhinitis can be divided into allergic rhinitis (AR) and non-allergic rhinitis (NAR). AR occurs when an allergen is the trigger for the nasal symptoms. NAR is when obstruction and rhinorrhea occurs in relation to non-allergic, noninfectious triggers, such as changes in the weather, cigarette smoke, etc [2]. Approximately 10%–25% of the world population is affected by AR [3]. The worldwide prevalence of allergic rhinitis among adults has been on the rise since at least 1990 [4]–[8]. In Sweden, the prevalence of AR increased from 22% to 31% from 1990 to 2008 in young adults [7]. However, less information is available on the prevalence or incidence of NAR. Generally, females [9], [10], young adults [10]–[12], and those with allergic heredity [13], [14] are at higher risk of having AR. Allergen sources in the home environment are common, such as from pets [15], cockroaches [12], [14], house dust mites, microbiologic agents [16], [17], etc. Signs of dampness and indoor molds is another common exposure that has been widely studied in relation to both AR and NAR in home [17]–[19]. Other factors such as traffic-related air pollution, different lifestyle and socioeconomic conditions are quoted as adjuvant factors for AR [20].

Asthma is the most common chronic respiratory disease in the world. Approximately 300 million people in the world currently have asthma [21]. Epidemiological studies have suggested that the prevalence of asthma in adults is approximately 7–10% in different parts of the western world [22]. The current trend (since 1990's) in the prevalence of asthma among adults is unclear, some studies suggesting that asthma prevalence has stabilized or even decreased [5], [7], [22], while, others suggesting it is still increasing [4]. However, asthma prevalence among children in many parts of the world is still increasing [23]. Asthma frequently co-exist with rhinitis [3], [24]–[26]. Adult asthma is more common among females [27]–[29], younger adults [27], and those with allergic heredity [27], [29]. Smoking [30], [31] and environmental tobacco smoke (ETS) [29], [32]–[34] were found to be associated with asthma or asthma related symptoms. A common finding is the association between observed building dampness and molds and adults' asthma [35]. Exposures related to building dampness can be house dust mites, molds, bacteria, and even chemicals (from the degradation process of building materials). Studies has shown that adults' asthma or asthmatic symptoms were associated with higher levels of cockroach allergen [36], mouse allergen [36], house dust mites [37], fungal [38], airborne bacteria [37], formaldehyde [39], [40] and total volatile organic compounds (VOC) [39], [40] in dwellings, newly painted indoor surfaces [41], and combustion of wood, coal or biomass fuels [34].

Respiratory infections include infections of the lower and upper respiratory tract, and otitis media [42]. Preventive strategies include attempting to avoid contact with or spreading of infectious agents and covering sneezes and vaccination for influenza and pneumococcal pneumonia [42]. The presence of building dampness and molds are associated with increase in respiratory infections in adults [19], [35], [42], [43]. Air movements in buildings and ventilation are essential for the transmission/spread of infectious diseases. There is some evidence of spread of infections by airborne transmission, such as measles, tuberculosis, chickenpox, influenza, smallpox and severe acute respiratory syndrome (SARS) [44]. There is however insufficient data to specify the minimum ventilation requirements to avoid the spread of infectious diseases via the airborne route [44], [45].

The aging population is an important issue in many countries [46]. A better understanding of the health consequences of exposure to different risk factors among elderly, notably to environmental factors, is needed. There are only few studies investigating the influence of environment factors among respiratory health in the elderly [47], [48]. Exposure to elevated levels of outdoor air pollution such as particle matter (PM), ozone (O_3_), nitrogen dioxide (NO_2_) and sulphur dioxide (SO_2_) were associated with increased hospital admissions for asthma and chronic obstructive pulmonary disease (COPD) [47]. For indoor environment, the most consistent association was found between increased risk of COPD and exposure to ETS [48].

The first aim of this study was to investigate the prevalence of rhinitis, asthma and respiratory infections among occupants living in multi-family buildings in Sweden. The second of aim was to study occupants' health symptoms in relation to their home environment. The third aim was to study if there is any difference of these associations in relation to occupants' age (comparing those less than or equal to 65 years old with those more than 65 years old).

## Material and Methods

### Ethics Statement

The study and the consent procedure were approved by the Regional Ethical Committee in Uppsala, Sweden. All participants gave informed consent. An information letter sent together with the questionnaire stated that if the subjects answered and returned the questionnaire it meant they had given informed consent.

### Selection of Buildings

The selection of multi-family buildings was performed by Statistics Sweden (SCB) [49]. The buildings were selected by a multi-stage sampling procedure with a first step to select 30 Swedish municipalities out of totally 290 municipalities across Sweden through a stratified random selection according to geographic and demographic characteristics (temperature zone and region), and degree of urbanization. The probability for a municipality to be selected was proportional to the size of the population of the municipality in December 31, 2006. The next step was selection of multi-family buildings from these 30 municipalities. Data on all multi-family buildings and their construction year were obtained from the central building register in Sweden. Stratified random sampling was used to sample buildings based on the construction year in 5 classes (before 1960, 1960–1975, 1976–1985, 1986–1995 and 1996–2005) aiming to get the same number of buildings in each age class. Since most buildings in Sweden are old, there was an over-sampling of new buildings in this study. Totally 690 multi-family buildings were sampled from the 30 municipalities.

### Questionnaire and study population

The study population consisted of all adults (≥18 years old) living in the multi-family buildings included in the study. There were totally 8841 apartments and 12488 adults. The questionnaires were developed at Department of Medical Science, Uppsala University, based on previous studies [7], [27], [50]–[53]. The number of adults living in each apartment was identified by SCB from the Swedish civil registration register. Each adult (≥18 years) registered in the selected apartments received a personal questionnaire including medicine questions and personal factors. Moreover, one indoor environment questionnaire was sent to each apartment. The postal questionnaire was administered by SCB during the spring 2008. Two reminders were sent to those who did not reply the first time. Totally, 5775 adults participated in the study and returned the medical questionnaire (46%) and 4369 indoor environment questionnaires (49%) were returned.

### Environmental questions

The home environment questionnaire included the following questions:

1. Number of persons living at home;
2. Ownership of the apartment (own/rented);
3. Type of ventilation system (mechanical ventilation system/only natural ventilation);
4. Current pet-keeping status in the apartment: dog (no/yes); cat (no/yes);
5. Whether anybody living in the apartment ride a horse or has in another way contact with horses (no/yes);
6. Current environmental tobacco smoke at home (ETS) (no, if the answer is “never”/yes, if the answer is “daily”, “weekly” or “monthly”);
7. Condensation on window panes in winter (no, if the answer is “never”/yes, if the answer is “less than 5 cm”, “5–25 cm” or “more than 25 cm”);
8. New indoor painting in the last 12 months (no/yes);
9. New floor materials in the last 12 months (no/yes);
10. Any water leakage in the last 12 months (no/yes);
11. Any floor dampness in the last 12 months (no/yes);
12. Any visible molds in the last 12 months (no/yes);
13. Any moldy odor in the last 12 months (no/yes);
14. Any other odor in the last 12 months (no/yes);
15. Any damp spot, visible mold or water leakage in the last 5 years (no/yes);
16. Any technical building investigation of the apartment by a consulting company because of dampness, water damage or mold (no/yes);

One new variable “dampness in the last 12 months” was created based on four questions (which are question (10) – (13)) among all the 16 questions above, named as question (17). The options for this variable are: no, if the answers for the four questions are all “no”; yes, if the answer of any of the four question is “yes”.

A dampness index was created based on question (15) and (17), which coded as: 0, both answers are “no”; 1, only one of the answers is “no”; 2, both answers are “yes”.

A window-opening index was created based on two questions about window opening habit as follows: a) How often do you usually air the apartment by opening the windows during heating season (from September to April)? There are four options: daily, weekly, monthly and never, which were coded as 1, 2, 3 and 4; b) if you do, how long time? There are four options: the whole day/night, few hours, have cross-draught for a few minutes and never, which were coded as 1, 2, 3 and 4. The window-opening index was coded as: 0, if b) = 4; 1, if a) = 3 and b) = 3; 2, if a) = 2 and b) = 3, or a) = 3 and b = 2, or a) = 1 b) = 3; 3, if a) = 2 and b) = 2; or if a) = 3 and b) = 1, or a) = 2 and b) = 1; 4, if a) = 1 and b) = 2; 5, if a) = 1 and b) = 1. A higher window-opening index indicate more window opening.

Data about construction year of buildings were collected from the National Building Register, Real Property Register and were categorized into 5 groups (-1960/1961–1975/1976–1985/1986–1995/1996–2005). Data about population and area of each municipality were acquired from wikipedia website (http://sv.wikipedia.org/wiki/Lista_%C3%B6ver_Sveriges_kommuner). Population density (number of persons per km^2^) were calculated based on population and area of each municipality. Data about temperature zone were collected from a Swedish government report [54], which has 4 catagories: 1, inner part of north Sweden (Norrland); 2, costal part of Norrland and some inner part of Svealand; 3, Svealand (main parts); 4, Göteland (main parts). A higher number indicate a warmer climate.

### Personal questions

Medical questions in the personal questionnaire included the following:

1. Gender (female/male);
2. Age;
3. Current rhinitis (Irritating, stuffy or runny nose in the last 3 months). There were three options: often (every week), sometimes, never;
4. Any wheezing in the chest in the last 12 months (no/yes);
5. Any daytime attack of time breathlessness at rest in the last 12 months (no/yes);
6. Any daytime attack of time breathlessness after exercise in the last 12 months (no/yes);
7. Woken by attack of time breathlessness at any time in the last 12 months (no/yes);
8. Diagnosed with asthma by a doctor (no/yes);
9. Asthma attack during the last 12 months (no/yes);
10. Current asthma medication (no/yes);
11. Number of respiratory infections (such as cold, throat infection or flu) in the last 3 months (no/yes)? The options were: never (no); 1 time and 2 or more times (yes);
12. Antibiotic medication because of respiratory infections during the last 12 months (no/yes);
13. Pollen allergy (no/yes);
14. Furry pet allergy (no/yes).

The variable daytime breathlessness was created from question (5) and (6) coded as “no” if the answers to both questions is “no”, and “yes” if any one of the answers is “yes”.

Current asthma was created from question (9) and (10) coded as “no” if the answers to both questions is “no”, and “yes” if any one of the answers is “yes”.

The variable pollen or furry pet allergy was created from question (13) and (14) coded as “no” if the answers to both questions is “no”, and “yes” if any one of the answers is “yes”.

The rhinitis variable was further classified into 4 categories: no rhinitis, infection rhinitis, allergic rhinitis and non-allergic rhinitis. Infection rhinitis was defined as having both current rhinitis and any respiratory infections in the last 3 months. Allergic rhinitis was defined as having current rhinitis and pollen of furry pet allergy, but no respiratory infections. Non-allergic rhinitis was defined as having current rhinitis, but no pollen or furry pet allergy and no respiratory infections.

### Statistical analysis

All statistical analyses were conducted with STATA 11.0 (STATA Corp, Texas, USA). The prevalence of symptoms and exposures were calculated for total subjects and age stratified groups. Ordinal regression models were used to study associations between current rhinitis, respiratory infections and home environment factors with adjustment for gender, age and current smoking. In the ordinal logistic regression OR is expressed for one step on the scale of the dependent variable. Mutual adjustment were then applied including all significant or nearly significant factors (p<0.1) with additional adjustment for gender, age and current smoking. As a next step, associations between different types of rhinitis symptoms and home environment factors were calculated by multi-nominal regression models with adjustment for gender, age and current smoking.

Multiple logistic regression was used to study associations between wheeze, day time breathlessness, night time breathlessness, doctor-diagnosed asthma, current asthma, respiratory infections and antibiotic medication for respiratory infections and home environment factors with adjustment for gender, age and smoking. Mutual adjustment were then applied including all significant or nearly significant factors (p<0.1) with additional adjustment for gender, age and current smoking.

In addition, sensitivity analyses were performed, by stratifying for age (≤65 years v.s >65 years old (elderly)).

Age interaction was studied in the mutual adjusted models (≤65 years old v.s elderly). Interaction was tested statistically if there were a marked numerical difference in OR when comparing the occupational active age group with elderly.

Associations were expressed as odds ratios (OR) with a 95% confidence interval (CI) for ordinal regression and logistic regression models. For multi-nominal regression models, associations were expressed as relative risk ratio (RRR) with a 95% confidence interval (CI). In all statistical analysis, two-tailed tests and a 5% level of significance were applied.

## Results

Totally, 5775 personal (46%) and 4369 indoor environment questionnaires (49%) were returned. 73 subjects did not fill the question of their gender, and 49 subjects did not fill the question of their of age. Analysis of non-responders was performed by SCB Sweden. SCB Sweden analyzed differences between participants and non-participants based on background factors available from other population registers linked to the census [54]. For privacy reasons we did not have access to all these variables. The participation rate was higher among older persons, especially those older than 60 years. Married persons had a higher participation rate, as compared to those not being married. Moreover, the participation rate was related to degree of urbanization, with a slightly lower participation rate in larger cities and suburban municipalities. The participation rate was slightly lower in older buildings. There was no major difference in participation rate between different municipalities. The differences in participation rate between those being born in Sweden as compared to those foreign-born persons and between those having or not having Swedish citizenship were very small. All of the differences in participation rate were small, except for age and civil status.

The prevalence of smoking, allergy, rhinitis, asthma and respiratory infection symptoms in relation to gender and different age groups are shown in Table 1. In total 56.5% were female, 63.5% were 18 to 65 years old, and 36.5% were more than 65 years old; 12.0% were current smokers; 51.0% had current rhinitis; 17.7% wheeze; 11.7% day time breathlessness; 6.0% night time breathlessness; 11.5% doctor diagnosed asthma; 7.7% current asthma; 46.4% respiratory infections and 11.9% antibiotic medication for respiratory infections. The prevalence of current smoking, pollen allergy, furry pet allergy, current rhinitis, asthma attack, respiratory infections were lower among elderly. Wheeze, day time breathlessness, night time breathlessness were higher among elderly.

**Table 1:** Data on gender, smoking, allergy, rhinitis, asthma and respiratory infections.

Category	Subcategory	Male n = 2483^b^ (%)	Female n = 3219^b^ (%)	*p* value^d^	≤65 years old n = 3637^c^ (%)	>65 years old n = 2089^c^ (%)	*p* value^d^	Total n = 5775 (%)
Female		-	-		54.2	60.4	<0.001	56.5
Current smoking		11.8	12.1	0.728	14.8	7.2	<0.001	12.0
Pollen allergy		23.9	23.7	0.891	28.3	15.7	<0.001	23.8
Furry pet allergy		12.2	11.6	0.505	15.5	5.4	<0.001	11.9
Pollen or furry pet allergy		27.4	27.1	0.847	32.7	17.6	<0.001	27.3
Rhinitis in the last 3 months	Never	49.4	48.7	<0.001	47.4	52.1	0.007	49.0
	Sometimes	41.3	37.2		40.3	36.7		39.1
	Weekly	9.3	14.1		12.3	11.2		11.9
Type of rhinitis	Never	49.6	49.0	0.308	47.6	52.4	<0.001	49.2
	Infection rhinitis	29.9	32.0		34.9	23.5		31.1
	Allergic rhinitis	8.2	7.3		8.3	6.5		7.7
	Non-allergic rhinitis	12.3	11.7		9.2	17.6		12.0
Wheeze in the last 12 months		17.3	17.8	0.642	16.4	19.9	0.001	17.7
Day time breathlessness in the last 12 months		10.8	12.3	0.078	10.3	14.0	<0.001	11.7
Night time breathlessness in the last 12 months		5.7	6.3	0.367	5.9	6.1	0.727	6.0
Doctor diagnosed asthma		9.9	12.6	0.002	12.0	10.3	0.056	11.5
Current asthma^a^		7.0	8.2	0.082	7.5	7.8	0.728	7.7
	Asthma attack	3.1	3.8	0.171	3.9	2.7	0.016	3.5
	Asthma medication	6.3	7.9	0.028	6.9	7.6	0.339	7.2
Respiratory infections in the last 3 months	No infection	53.1	53.9	0.191	48.0	63.1	<0.001	53.6
	One time	36.2	34.3		38.4	29.6		35.1
	Two or more times	10.6	11.8		13.6	7.3		11.3
Used antibiotic medication for respiratory infections in the last 12 months		9.7	13.5	<0.001	11.2	12.9	0.058	11.9

a Subjects who have had asthma attacks or have taken asthma medicine during the last 12 months.

b Subjects with missing data on gender (n = 73) were excluded.

c Subjects with missing data on age (n = 49) were excluded.

d *p* value by Chi-square test.

Descriptive data on home environment factors are shown in Table 2. Totally, 40.9% were living alone; 50.7% were living in rented apartments; 19.0% had window pane condensation; 14.7% had any dampness in the last 12 months and 19.3% in the last 5 years. Females were more living in rented apartments and more often in certain building years. Compared with subjects ≤65 years old, there were more elderly were living alone. The elderly had less cats, and less contact with horses, less exposure to ETS, less renovation (painting, new floors) and less dampness, visible molds and odors.

**Table 2:** Data on home building characteristics and indoor environment factors.

Home environment factors	Subcategory	Male n = 2483^e^ (%)	Female n = 3219^e^ (%)	*p* value^g^	≤65 years old n = 3637^f^ (%)	>65 years old n = 2089^f^ (%)	*p* value^g^	Total n = 5775 (%)
Temperature zone^a^	1	2.6	2.7	0.967	2.5	2.8	0.016	2.6
	2	11.0	11.1		10.1	12.6		11.0
	3	52.6	51.9		53.2	50.4		52.1
	4	33.9	34.4		34.2	34.1		34.3
Construction year	-1960	12.9	12.1	0.017	13.6	10.5	<0.001	12.5
	1961–1975	33.7	32.4		34.0	31.0		33.0
	1976–1985	16.8	18.6		17.0	19.1		17.9
	1986–1995	14.6	17.0		15.1	17.5		16.0
	1996–2005	21.9	19.8		20.2	21.9		20.7
Living alone		41.0	40.9	0.991	36.6	48.4	<0.001	40.9
Rented apartments		49.1	52.0	0.031	52.7	47.0	<0.001	50.7
Natural ventilation only		45.7	45.3	0.788	46.5	43.6	0.063	45.4
Have dog		7.5	7.7	0.726	8.1	6.9	0.112	7.7
Have cat		8.0	9.1	0.166	9.7	6.7	<0.001	8.7
Horse contact		3.2	3.6	0.376	4.4	1.8	<0.001	3.5
ETS		11.9	12.0	0.928	13.1	10.2	0.003	12.1
New indoor painting in the last 12 months		18.0	16.3	0.088	19.1	13.6	<0.001	17.1
New floor materials in the last 12 months		9.6	9.5	0.866	10.8	7.4	<0.001	9.5
Window pane condensation		19.4	18.7	0.535	21.3	15.2	<0.001	19.0
Water leakage in the last 12 months		7.1	6.4	0.306	7.4	5.4	0.004	6.7
Floor dampness in the last 12 months		6.3	7.0	0.287	7.6	5.1	<0.001	6.8
Visible molds in the last 12 months		4.0	3.5	0.355	4.5	2.4	<0.001	3.8
Moldy odor in the last 12 months		2.6	2.7	0.799	3.2	1.5	<0.001	2.7
Other odor in the last 12 months		8.2	8.7	0.611	9.2	7.2	0.026	8.5
Any dampness in the last 12 months^b^		15.0	14.4	0.536	16.4	11.4	<0.001	14.7
Any dampness in the last 5 years		20.1	18.5	0.156	21.3	15.3	<0.001	19.3
Consultant investigation for home dampness		15.4	13.9	0.177	15.8	12.4	0.003	14.5
Dampness index^c^	0	74.1	75.4	0.514	72.3	79.5	<0.001	74.7
	1	16.1	15.7		17.1	13.7		15.9
	2	9.7	9.0		10.6	6.9		9.3
Window-opening index^d^	0	2.0	1.8	0.912	2.2	1.4	<0.001	1.9
	1	2.9	2.6		3.1	2.0		2.7
	2	23.4	23.1		24.2	21.8		23.2
	3	11.2	10.8		11.9	9.3		11.0
	4	36.8	37.0		34.6	41.0		37.1
	5	23.5	24.6		23.9	24.5		24.2

a Higher number stands for warmer climate.

b Subjects have reported one of the following dampness problems: water leakage, floor dampness, visible molds or moldy odor in the last 12 months.

c Dampness index, number of “yes” answers to “any dampness in the last 12 months” and “any dampness in the last 5 year”: 0, answers were both “no”; 1, only one answer was “yes”; 2, answers were “yes” to both questions.

d Higher number stands for more window opening.

e Subjects with missing data on gender (n = 73) were excluded.

f Subjects with missing data on age (n = 49) were excluded.

g *p* value by Chi-square test.

The associations between current rhinitis and home environment factors are shown in Table 3 (mutual adjustment). Current rhinitis was more common in those living in buildings constructed during 1976–1985, as compared to older buildings constructed before 1961 (reference category). Moreover, living in densely populated areas, living alone and living in rented apartments were associated with current rhinitis.

**Table 3:** Associations between current rhinitis and building characteristics and indoor environment factors analyzed by ordinal regression models.

Factors	Subcategory	All subjects n = 5775 OR(95% CI)^c^	All subjects n = 5775 Mutual adj. OR(95% CI)^d^	≤65 years old, n = 3637^g^, Mutual adj. OR(95% CI)^e^	>65 years old n = 2089^g^, Mutual adj. OR(95% CI)^f^
Construction year	-1960	1.00	1.00	1.00	1.00
	1961–1975	1.23(1.03,1.46)*	1.12(0.90,1.41)	1.17(0.90,1.52)	1.12(0.73,1.72)
	1976–1985	1.34(1.10,1.63)**	1.43(1.12,1.84)**	1.45(1.07,1.96)*	1.54(0.97,2.46)
	1986–1995	1.18(0.96,1.44)	1.27(0.99,1.63)	1.30(0.96,1.74)	1.32(0.83,2.11)
	1996–2005	1.01(0.83,1.22)	1.15(0.91,1.46)	1.24(0.93,1.64)	1.12(0.71,1.76)
Population density/10000^a^		1.48(1.12,1.97)**	1.61(1.13,2.29)**	1.74(1.14,2.64)*	1.81(0.90,3.64)
Living alone		1.14(1.02,1.27)*	1.25(1.09,1.43)**	1.54(1.30,1.82)***	0.88(0.69,1.11)
Rented apartments		1.33(1.20,1.49)***	1.23(1.07,1.40)**	1.25(1.06,1.48)**	1.21(0.95,1.54)
Window pane condensation		1.23(1.07,1.42)**	1.16(0.98,1.38)	1.15(0.94,1.41)	1.22(0.88,1.68)
Odor, other than moldy odorin the last 12 months		1.21(0.98,1.50)	1.25(0.98,1.60)	1.30(0.97,1.73)	1.03(0.63,1.67)
Dampness index^b^	0	1.00	1.00	1.00	1.00
	1	1.10(0.94,1.28)	1.07(0.88,1.29)	1.32(1.05,1.67)*	0.67(0.46,0.98)*
	2	1.26(1.04,1.53)*	1.19(0.88,1.60)	1.46(1.03,2.06)*	0.64(0.34,1.21)

a The ORs were expressed per 10000 increase of the population density (number of persons per km^2^).

b Dampness index, number of “yes” answers to “any dampness in the last 12 months” and “any dampness in the last 5 year”: 0, answers were both “no”; 1, only one answer was “yes”; 2, answers were “yes” to both questions.

c Adjusted for gender, age and smoking.

d Mutual adjustment models including gender, age and smoking for all subjects (inclusion criteria is *p*<0.1 in total sample).

e Mutual adjustment models including gender, age and smoking for younger and middle aged subjects (≤65 years old) (inclusion criteria is *p*<0.1 in total sample).

f Mutual adjustment models including gender, age and smoking for elderly (>65 years old) (inclusion criteria is *p*<0.1 in total sample).

g Subjects with missing data on age (n = 49) were excluded.

*** *p*<0.001,

** *p*<0.01,

* *p*<0.05.

When dividing type of rhinitis into three groups (infection- allergic- and non-allergic rhinitis), infection rhinitis was more common among those living in buildings constructed during 1976–1985, as compared with older buildings constructed before 1961 and other buildings constructed after 1985. Moreover, it was more common in densely populated areas, among those living alone, those in rented apartments and in homes with window condensation in winter time. Living alone and window pane condensation were risk factors for allergic rhinitis. Non-allergic rhinitis was more reported among those living in rented apartments (Tables 4).

**Table 4:** Associations between different types of rhinitis symptoms and building characteristics and indoor environment factors analyzed by multi-nominal logistic regression models.

Factors	Infection rhinitis n = 1568 RRR(95% CI)^a^	Allergic rhinitis n = 388 RRR(95% CI)^a^	Non-allergic rhinitis n = 605 RRR(95% CI)^a^
Construction year 1961–1975^b^	1.09(0.94,1.27)	1.16(0.91,1.48)	1.21(0.99,1.48)
Construction year 1976–1985^c^	1.32(1.11,1.57)**	0.93(0.68,1.28)	1.10(0.86,1.42)
Population density^d^	1.24(1.09,1.42)**	1.08(0.86,1.34)	1.00(0.84,1.20)
Living alone	1.16(1.02,1.33)*	1.37(1.10,1.69)**	1.03(0.85,1.23)
Rented apartments	1.28(1.12,1.46)***	1.16(0.93,1.45)	1.35(1.13,1.63)**
Window pane condensation	1.24(1.04,1.47)*	1.32(1.00,1.75)*	1.13(0.89,1.45)
Dampness index = 2^e^	1.21(0.96,1.53)	1.41(0.98,2.03)	0.98(0.69,1.40)

a Adjusted for gender, age and smoking.

b Buildings constructed during 1961–1975 compared with buildings constructed before 1960 and during 1986–2005 (excluding buildings constructed during 1976–1985).

c Buildings constructed during 1976–1985 compared with buildings constructed before 1960 and during 1986–2005 (excluding buildings constructed during 1961–1975).

d Population density (number of persons per km^2^) was used as a categorical variable in this analysis: population density >1000 persons/km^2^ was compared with population density ≤1000 persons/km^2^.

e Dampness index, number of “yes” answers to “any dampness in the last 12 months” and “any dampness in the last 5 year”: 0, answers were both “no”; 1, only one answer was “yes”; 2, answers were “yes” to both questions. Dampness index  = 2 was compared with dampness index  = 0 in this analysis.

*** *p*<0.001,

** *p*<0.01,

* *p*<0.05.

The associations between wheeze, day time breathlessness, night time breathlessness and home environment factors are shown in Table 5 (mutual adjustment). Wheeze was more common among those living in colder climate zones, in rented apartments and in homes with dampness problems (dampness index). Day time attacks of breathlessness was associated with living in buildings constructed during 1961–1975, in rented apartments, in homes with dampness problems, and in apartments with more window-opening (high window-opening index). The association between day time breathlessness and dampness close to significance for dampness index = 1 (*p* = 0.05). Night time breathlessness was more reported in colder climate zones and in apartments with more window-opening time. The associations between doctor-diagnosed asthma, current asthma and home environment factors are shown in Table 6 (mutual adjustment). Doctor-diagnosed asthma was associated with living in homes with odor, other than moldy odor. Current asthma was more common in homes with ETS and in homes with odor, other than moldy odor.

**Table 5:** Associations between wheeze, day time breathlessness, night time breathlessness, building characteristics and indoor environment factors (by logistic regresson models).

Symptoms	Factors	Subcategory	All subjects n = 5775 OR(95% CI)^d^	All subjects n = 5775, mutual adj. OR(95% CI)^e^	≤65 years old n = 3637^h^, mutual adj. OR(95% CI)^f^	>65 years old n = 2089^h^, mutual adj. OR(95% CI)^g^
Wheeze in the last 12 months	Temperature zone^a^		0.91(0.83,1.01)	0.89(0.80,0.99)*	0.87(0.75,1.00)*	0.90(0.76,1.06)
	Rented apartments		1.35(1.16,1.56)***	1.20(1.02,1.41)*	1.10(0.89,1.35)	1.37(1.06,1.77)*
	Window pane condensation		1.20(0.99,1.43)	1.15(0.95,1.41)	1.13(0.89,1.44)	1.17(0.83,1.65)
	Dampness index^b^	0	1.00	1.00	1.00	1.00
		1	1.16(0.95,1.42)	1.06(0.85,1.33)	1.27(0.96,1.67)	0.77(0.52,1.15)
		2	1.53(1.20,1.94)**	1.42(1.08,1.86)*	1.90(1.38,2.62)***	0.69(0.38,1.22)
Day time breathlessness in the last 12 months	Construction year	-1960	1.00	1.00	1.00	1.00
		1961–1975	1.37(1.03,1.83)*	1.53(1.03,2.29)*	1.47(0.93,2.33)	2.13(0.91,4.99)
		1976–1985	1.25(0.91,1.72)	1.28(0.82,2.01)	1.11(0.65,1.91)	2.20(0.89,5.45)
		1986–1995	1.20(0.87,1.66)	1.39(0.89,2.18)	1.38(0.81,2.36)	1.94(0.78,4.81)
		1996–2005	0.76(0.54,1.05)	1.12(0.71,1.76)	0.95(0.55,1.63)	1.92(0.78,4.71)
	Rented apartments		1.60(1.34,1.90)***	1.31(1.04,1.66)*	1.25(0.93,1.69)	1.45(0.99,2.12)
	Natural ventilation only		1.35(1.12,1.63)**	1.13(0.90,1.42)	1.04(0.77,1.39)	1.47(1.01,2.14)*
	ETS		1.32(1.03,1.70)*	1.15(0.83,1.57)	1.03(0.70,1.54)	1.41(0.82,2.41)
	New indoor painting in the last 12 months		0.79(0.62,1.00)	0.71(0.50,1.01)	0.70(0.45,1.07)	0.76(0.39,1.47)
	New floor materials in the last 12 months		0.75(0.54,1.03)	0.62(0.38,1.00)	0.61(0.35,1.08)	0.62(0.24,1.55)
	Dampness index^b^	0	1.00	1.00	1.00	1.00
		1	1.35(1.07,1.70)*	1.35(1.00,1.81)*	1.44(1.00,2.07)	1.26(0.76,2.10)
		2	1.76(1.34,2.30)***	1.57(1.09,2.27)*	2.18(1.44,3.31)***	0.48(0.19,1.25)
	Window-opening index^c^		1.08(1.01,1.16)*	1.10(1.01,1.20)*	1.06(0.95,1.19)	1.20(1.02,1.40)*
Night time breathlessness in the last 12 months	Temperature zone^a^		0.85(0.74,0.99)*	0.74(0.61,0.90)**	0.76(0.59,0.97)*	0.69(0.50,0.97)*
	Construction year	-1960	1.00	1.00	1.00	1.00
		1961–1975	1.65(1.11,2.44)*	1.19(0.70,2.01)	1.39(0.76,2.57)	0.93(0.32,2.67)
		1976–1985	1.44(0.93,2.22)	1.23(0.69,2.18)	1.18(0.59,2.35)	1.54(0.51,4.64)
		1986–1995	1.42(0.91,2.22)	1.04(0.57,1.89)	0.95(0.45,1.99)	1.39(0.47,4.15)
		1996–2005	0.64(0.39,1.05)	0.55(0.29,1.05)	0.53(0.24,1.17)	0.63(0.19,2.06)
	Rented apartments		1.66(1.31,2.09)***	1.34(0.98,1.84)	1.27(0.85,1.89)	1.51(0.87,2.62)
	Natural ventilation only		1.25(0.97,1.62)	0.99(0.72,1.35)	1.02(0.69,1.50)	0.99(0.56,1.73)
	New indoor painting in the last 12 months		0.68(0.49,0.96)*	0.64(0.40,1.01)	0.53(0.31,0.92)*	0.95(0.42,2.19)
	Window pane condensation		1.35(1.02,1.80)*	1.10(0.76,1.59)	1.01(0.65,1.58)	1.28(0.64,2.55)
	Dampness index^b^	0	1.00	1.00	1.00	1.00
		1	1.41(1.04,1.90)*	1.10(0.72,1.68)	1.27(0.77,2.09)	0.87(0.39,1.92)
		2	1.46(1.01,2.13)*	1.60(0.96,2.64)	2.63(1.46,4.36)**	-
	Window-opening index^c^		1.12(1.02,1.24)*	1.18(1.04,1.34)*	1.13(0.97,1.32)	1.26(0.99,1.61)

a The ORs were expressed per 1 unit increase for temperature zone.

b Dampness index, number of “yes” answers to “any dampness in the last 12 months” and “any dampness in the last 5 year”: 0, answers were both “no”; 1, only one answer was “yes”; 2, answers were “yes” to both questions.

c The ORs were expressed per 1 unit increase for window-opening index.

d Adjusted for gender, age and smoking.

e Mutual adjustment models including gender, age and smoking for all subjects (inclusion criteria is *p*<0.1 in total sample).

f Mutual adjustment models including gender, age and smoking for subjects ≤65 years old (inclusion criteria is *p*<0.1 in total sample).

g Mutual adjustment models including gender, age and smoking for subjects >65 years old (inclusion criteria is *p*<0.1 in total sample).

h Subjects with missing data on age (n = 49) were excluded.

*** *p*<0.001,

** *p*<0.01,

* *p*<0.05.

**Table 6:** Associations between doctor-diagnosed asthma, current asthma, building characteristics and indoor environment factors analyzed by logistic regression models.

Symptoms	Factors	Subcategory	All subjects n = 5775 OR(95% CI)^d^	All subjects n = 5775, mutual adj. OR(95% CI)^e^	≤65 years old n = 3637^h^, mutual adj. OR(95% CI)^f^	>65 years old n = 2089^h^, mutual adj. OR(95% CI)^g^
Doctor-diagnosed asthma	Temperature zone^b^		0.90(0.80,1.01)	0.95(0.83,1.08)	0.86(0.74,1.01)	1.16(0.90,1.50)
	Rented apartments		1.24(1.04,1.47)*	1.13(0.93,1.38)	0.97(0.76,1.23)	1.69(1.15,2.46)**
	Odor, other than moldy odor in the last 12 months		1.52(1.12,2.06)**	1.49(1.08,2.06)*	1.66(1.15,2.39)**	1.04(0.50,2.16)
	Dampness index^c^	0	1.00	1.00	1.00	1.00
		1	1.10(0.87,1.39)	1.14(0.87,1.49)	1.32(0.98,1.80)	0.67(0.36,1.25)
		2	1.34(1.01,1.78)*	1.20(0.79,1.81)	1.48(0.95,2.33)	0.50(0.15,1.66)
Current asthma^a^	Temperature zone^b^		0.89(0.78,1.02)	0.90(0.75,1.06)	0.78(0.64,0.95)*	1.25(0.90,1.74)
	Construction year	-1960	1.00	1.00	1.00	1.00
		1961–1975	1.17(0.84,1.62)	1.08(0.72,1.63)	0.99(0.62,1.58)	1.32(0.53,3.27)
		1976–1985	1.10(0.76,1.58)	1.02(0.64,1.62)	0.96(0.56,1.64)	1.26(0.47,3.35)
		1986–1995	1.09(0.75,1.59)	0.88(0.55,1.42)	0.73(0.42,1.28)	1.29(0.49,3.40)
		1996–2005	0.70(0.48,1.03)	0.70(0.43,1.13)	0.75(0.44,1.30)	0.60(2.12,1.72)
	Rented apartment		1.32(1.07,1.62)**	1.05(0.81,1.36)	0.95(0.69,1.29)	1.45(0.90,2.32)
	ETS		1.43(1.06,1.92)*	1.53(1.09,2.16)*	1.43(0.94,2.17)	1.79(0.97,3.34)
	New indoor painting in the last 12 months		0.73(0.54,0.98)*	0.69(0.47,0.99)*	0.69(0.45,1.04)	0.61(0.27,1.37)
	Odor, other than moldy odor in the last 12 months		1.64(1.15,2.33)**	1.52(1.03,2.24)*	1.71(1.10,2.65)*	1.05(0.43,2.52)
	Dampness index^c^	0	1.00	1.00	1.00	1.00
		1	1.14(0.86,1.52)	1.29(0.93,1.79)	1.57(1.07,2.29)*	0.76(0.37,1.58)
		2	1.50(1.08,2.08)*	1.14(0.68,1.92)	1.62(0.93,2.82)	0.22(0.03,1.63)

a subjects who have had asthma attacks or have taken asthma medicine during the last 12 months.

b The ORs were expressed per 1 unit increase for temperature zone.

c Dampness index, number of “yes” answers to “any dampness in the last 12 months” and “any dampness in the last 5 year”: 0, answers were both “no”; 1, only one answer was “yes”; 2, answers were “yes” to both questions.

d Adjusted for gender, age and smoking.

e Mutual adjustment models including gender, age and smoking for all subjects (inclusion criteria is *p*<0.1 in total sample).

f Mutual adjustment models including gender, age and smoking for subjects ≤65 years old (inclusion criteria is *p*<0.1 in total sample).

g Mutual adjustment models including gender, age and smoking for subjects >65 years old (inclusion criteria is *p*<0.1 in total sample).

h Subjects with missing data on age (n = 49) were excluded.

*** *p*<0.001,

** *p*<0.01,

* *p*<0.05.

The associations between respiratory infections, used antibiotic medication for respiratory infections and home environment factors are shown in Table 7 (mutual adjustment). Respiratory infections were associated with living in buildings constructed during 1976–1985, as compared with older buildings constructed before 1960, and in densely populated areas and in rented apartments. Antibiotic medication for respiratory infections was more common in homes with window pane condensation.

**Table 7:** Associations between respiratory infections (by ordinal regression models), antibiotic medication for respiratory infections (by logistic regression models), building characteristics and indoor environment factors.

Symptoms	Factors	Subcategory	All subjects n = 5775 OR(95% CI)^d^	All subjects n = 5775, mutual adj. OR(95% CI)^e^	≤65 years old n = 3637^h^, mutual adj. OR(95% CI)^f^	>65 years old n = 2089^h^, mutual adj. OR(95% CI)^g^
Respiratory infections in the last 3 months	Construction year	-1960	1.00	1.00	1.00	1.00
		1961–1975	1.06(0.89,1.26)	1.07(0.90,1.28)	1.07(0.87,1.31)	1.11(0.79,1.56)
		1976–1985	1.41(1.17,1.71)***	1.46(1.21,1.78)***	1.33(1.06,1.68)*	1.87(1.30,2.69)**
		1986–1995	1.07(0.88,1.30)	1.08(0.88,1.31)	1.03(0.81,1.31)	1.25(0.86,1.80)
		1996–2005	1.09(0.91,1.31)	1.12(0.93,1.35)	0.97(0.78,1.21)	1.56(1.09,2.22)*
	Population density^a^		1.57(1.19,2.09)**	1.72(1.30,2.29)***	1.68(1.20,2.34)**	2.21(1.27,3.87)**
	Rented apartments		1.11(1.00,1.23)	1.13(1.01,1.26)*	1.13(0.99,1.29)	1.14(0.94,1.37)
Antibiotic medication for respiratory infections in the last 12 months	Population density^a^		1.67(1.08,2.58)*	1.63(0.94,2.85)	1.96(0.99,3.90)	1.53(0.58,4.09)
	Rented apartments		1.22(1.03,1.44)*	1.13(0.91,1.40)	1.46(1.10,1.92)**	0.84(0.59,1.20)
	Window pane condensation		1.33(1.08,1.64)**	1.41(1.10,1.82)**	1.49(1.09,2.03)*	1.23(0.78,1.94)
	Odor, other than moldy odor in the last12 months		1.42(1.05,1.92)*	1.23(0.85,1.80)	1.20(0.75,1.91)	1.22(0.64,2.35)
	Dampness index^b^	0	1.00	1.00	1.00	1.00
		1	1.25(1.00,1.57)	1.08(0.80,1.47)	1.29(0.90,1.86)	0.77(0.42,1.39)
		2	1.43(1.08,1.89)*	1.40(0.92,2.12)	1.84(1.14,2.97)*	0.63(0.24,1.64)
	Window-opening index^c^		1.06(0.99,1.14)	1.09(1.00,1.18)	1.05(0.95,1.17)	1.13(0.97,1.31)

a The ORs were expressed per 10000 increase of the population density (number of persons per km^2^).

b Dampness index, number of “yes” answers to “any dampness in the last 12 months” and “any dampness in the last 5 year”: 0, answers were both “no”; 1, only one answer was “yes”; 2, answers were “yes” to both questions.

c The ORs were expressed per 1 unit increase for window-opening index.

d Adjusted for gender, age and smoking.

e Mutual adjustment models including gender, age and smoking for all subjects (inclusion criteria is *p*<0.1 in total sample).

f Mutual adjustment models including gender, age and smoking for subjects ≤65 years old (inclusion criteria is *p*<0.1 in total sample).

g Mutual adjustment models including gender, age and smoking for subjects >65 years old (inclusion criteria is *p*<0.1 in total sample).

h Subjects with missing data on age (n = 49) were excluded.

*** *p*<0.001,

** *p*<0.01,

* *p*<0.05.

Among elderly, current rhinitis was less common among those in homes with dampness problems. Wheeze was more common in rented apartments. Day time attacks of breathlessness was associated with living in homes without mechanical ventilation and more common in homes with high window-opening index. Night time attacks of breathlessness was more reported among those living in colder climate zones. Doctor-diagnosed asthma was more common among those living in rented apartments. Respiratory infections was associated with living in buildings constructed during 1976–1985 and 1996–2005, as compared to older buildings constructed before 1961, and it was more commonly reported in less populated areas. Generally, for these associations OR were numerically higher among elderly as compared to subjects ≤65 years old.

Finally, statistical interaction was tested, comparing elderly with subjects ≤65 years old. There was an age interaction for the association between dampness index and the following health variables: current rhinitis (for interaction, p = 0.001), wheeze (for interaction, p = 0.001), day time attacks of breathlessness (for interaction, p = 0.002), night time attacks of breathlessness (for interaction, p = 0.004), doctor-diagnosed asthma (for interaction, p = 0.024), current asthma (for interaction, p = 0.021) and antibiotic medication for respiratory infections (for interaction, p = 0.015). There was a trend for age interaction between apartment ownership and doctor-diagnosed asthma (for interaction, p = 0.056). Moreover, there was an age interaction for the association between ownership and antibiotic medication for respiratory infections (for interaction, p = 0.015).

## Discussions

Associations were demonstrated between home environmental factors in multi-family buildings and adults' rhinitis, asthma and respiratory symptoms. Densely populated areas (urbanization), colder climate zones, rented apartments, building dampness, certain construction years (1961–1975, 1976–1985), odor perception and ETS at home were risk factors for rhinitis, asthma symptoms and respiratory infections.

This study was made during the spring season, which is part of the pollen season in Sweden. Selection bias due to a low response rate can influence epidemiological studies. In this study, we included all subjects (≥18 years old) living in multi-family buildings, with no prior information on their health status. The sample size of this study was reasonably large, and is one of few covering a sample from a whole country. The response rate was not very high (46%). The participation rate was higher among elderly persons, married persons, and slightly lower in larger cities, suburban municipalities and in older buildings. However, we have found no major difference in participation rate between different municipalities, between those being born in Sweden as compared to those foreign-born persons, and between those having or not having Swedish citizenships. Furthermore, most of the differences in participation rate were small. The elderly in our study accounted for 36% in our study. It has been shown that elderly population (more than 65 years old) in Sweden are accounting for 23% of the adult population [55]. Elderly in our study were more prone to answer questionnaire, and retired people lives more in apartments other than in single-family buildings in Sweden. Recall bias can be a potential problem in questionnaire surveys. Subjects may overestimate or underestimate their personal symptoms or reports on indoor environment risk factors. However, some factors, such as building constructed year, population density were not collected by questionnaire. Moreover, we found some associations between specific home environmental risk factors and health status, not a similar association for all risk factors. Thus, we don't believe that our conclusions are seriously biased by selection or response errors.

Our study found that subjects living in colder climate zones reported more wheeze and night time breathlessness. This is in agreement with a previous study reported more asthma among Swedish conscripts in the colder parts of northern Sweden [56]. One possible explanation could be less ventilation to save energy.

Living in an area with a higher population density (persons/km^2^) was associated with current rhinitis, infection rhinitis and respiratory infections. One study in Swedish preschool children found that asthma prevalence increased by municipality population density measured the same way as in our study [57]. Population density is used as a proxy variable for degree of urbanization. Exposure to traffic air pollution is one factor associated with population density. Outdoor traffic related exposure was related to an increased risk of allergic rhinitis among adults in Italy [58] and in Sweden [59]. The risk for having infections would increase with the increase of population density because of increased probability of spending time in crowded environments.

Despite the low prevalence of ETS in the Swedish homes, we confirmed that ETS at home is still a risk factor for current asthma. ETS in homes is one of the most consistently factors associated with asthma and asthma-related symptoms among adults in homes [34].

Subjects renting their apartment had more current rhinitis, infection rhinitis, wheeze, day time breathlessness and respiratory infections. Renting apartment can be a proxy variable for lower socio-economic status (SES). One Swedish study on multi-family buildings in Stockholm found that dwellers renting their apartments reported more nasal symptoms than those owning their homes, and found a high correlation between different socio-economic factors (apartment ownership, income, education, and occupation) [60]. One study on SES (defined by parental education) and children's respiratory and allergic symptoms conducted in 13 diverse countries found that lower SES was related to decreased prevalence of allergy to aero-allergens [61]. The association between SES and asthma prevalence and incidence in adults is not well understood and is a matter of debate [62]. However, lower SES seems to be associated with more severe asthma or poorly controlled asthma [62]. Asthma prevalence was higher in lower SES groups among adults [63] and among children [61]. In Sweden, a large number of multi-family apartments of all construction periods are rental, owned by the community, with similar living standards and rental costs as private apartments [64]. However families with lower SES are overrepresented in buildings built from 1961–1975, mostly rented and today buildings prioritized for renovation. The influence of ownership on health in our study may also depend upon differences in building maintenance, e.g. a lack of control over the home environment because of renting rather than owning.

Our study found that reported building dampness (dampness index) was associated with wheeze and day time breathlessness. This finding is consistent with another Swedish study among adults living in multi-family buildings [65]. Building dampness problems in homes is associated with increased asthma prevalence, which has been shown in former studies [35], [43], [66], [67]. A cohort study found that new onset of adult asthma was related to dampness/molds in dwellings in Europe, and concluded that the population-attributable risk was 3–14% for observed dampness/molds [68]. One meta-analysis showed that wheeze among adults were associated with damp homes, and concluded that building dampness and molds were associated with approximately 30–50% increases in a variety of respiratory and asthma-related health outcomes [43]. Building dampness is related to a complex mix of exposures, including house dust mite allergens [69], microbial agents [70], [71], bacteria [71]–[73], chemical emissions from degradation of building materials [74] as well as microbial volatile organic compounds (MVOC) [71].

Window pane condensation was associated with infection rhinitis, allergic rhinitis and antibiotic medication for respiratory infections. Window pane condensation is usually a marker of poor ventilation and a predictor of high relative air humidity (RH) indoors. A Japanese study found that window pane condensation in homes of female university students was related to wheeze symptoms [75]. Exhaled nitric oxide (NO) levels were associated with home window pane condensation among non-sensitized (skin prick tests for IgE sensitization) school children aged [76]. NO is a method introduced for non-invasive monitoring of airway inflammation.

We found that certain construction periods were associated with more respiratory illness. Buildings constructed from 1961–1975 were associated with more day time breathlessness. Buildings constructed from 1976–1985 were associated with more current rhinitis, infection rhinitis and respiratory infections in the last 3 months. Our age classification of multi-family buildings were based on major changes in buildings technology in Sweden. Buildings from 1961–1975 were constructed during a building boom in suburbs of large cities rented by poorer people. They were mostly poor quality high-rise buildings with basements and only exhaust ventilation. These buildings have been reported to have the highest prevalence of dampness problems [77]. The next generation of multi-family buildings (constructed during 1976–1985) was influenced by energy saving demands, with many changes in construction techniques and new building materials [64]. The drastic increase of the oil price in 1974 initiated energy saving measures to reduce energy consumption in buildings during this period. Moreover, in 1977 self–level mortar containing the protein casein were introduced and used in Swedish buildings from 1977–1983. Due to severe indoor environmental problems this mortar was not used after 1983. Studies have shown that the problem was due to dampness causing chemical emissions such as ammonia and sulfhydryl compounds [78]. Later research identified another odorous compound, 2-acetophenone, emitted from the casein containing mortar. The compound is produced by degradation of the amino acid tryptophan [79]. It has a raw stuffy smell, and has been identified as a compound causing malodour in wine [80] and green tea [81]. The emission of ammonia from the mortar is known to cause blackening of parquet floors and degradation of acrylate polymers in water based glue and the plasticizer di-di-ethyl-hexyl-phthlate (DEHP) in polyvinylchloride (PVC) floor materials, causing emission of 2-etyl-1-hexanol to indoor air [82]. Epidemiological studies have shown that subjects staying in buildings with this type of mortar, and with emission of ammonia and 2-etyl-1-hexanol have a higher prevalence of mucosal inflammation and sick building syndrome [82] and asthma [74]. Thus, multifamily buildings constructed 1976-1984 had particular indoor exposures and were the first generation of buildings in Sweden with low ventilation due to increased energy saving demand and testing of new building materials on a large scale. This may have resulted in an impaired indoor environment leading to the elevated levels of health problems observed in this study.

More window-opening time (higher window-opening index) was associated increased risk of day time breathlessness and night time breathlessness. A review article concluded that higher ventilation rate was associated with less report of asthma or asthma-related symptoms [45]. The negative association between window-opening time and respiratory symptoms in our study is unclear. Polluted outdoor air entering the indoor environment when opening windows could be one explanation. However, this study included all multi-family buildings of the whole country, not only a small polluted area of Sweden. Another possible reason would be that those with asthma problems tried to improve ventilation situation by opening window more often (limitation of the cross sectional design).

We found associations between other odor perception (moldy odor was included in the dampness index) and doctor-diagnosed asthma and current asthma. Experimental studies have shown that odor thresholds are similar in asthmatic and non-asthmatic subjects [83]. Negative or positive attitudes to the odors may (cognitive bias) influence the occurrence of irritative symptoms on experimental exposure to odorous compounds [84]. As our results were based on mutual adjustment models, it is less likely that differences in personal factors or attitudes would have biased our results. Odor perception in our study may be an indicator of poor ventilation. Increased ventilation was found to be associated with less report of odor among university students in computer classrooms [85]. Reported odor was related to higher levels of total fungal DNA in Swedish daycare centers in Sweden [86]. Pungent odor, musty odor and stuffy odor in multi-family buildings in Sweden were associated with asthma symptoms among adults [65]. Self-report of odor perceptions in dwellings were also found to be associated with SBS among adults in China [87]. Thus, odor perception can be associated with different types of exposure and can be of importance for asthma.

New indoor painting in the last 12 months was negatively associated with current asthma, and there was a same trend for its association with night time breathlessness in our study. One former study found that self-reported domestic exposure to newly painted surfaces in the last 12 months in Swedish homes was positively associated with adults' nocturnal breathlessness [41]. The reason for the opposite finding in our study is not clear. Maybe some other activities that related to indoor painting improved the indoor environment but was not measured in our study. Paint used in indoor environment has becoming more and more environmental friendly with less and less chemical emissions, especially in developed countries like Sweden. One study compared new paint and conventional paint and found a significant increase in wheeze and breathlessness during use of conventional paint, but not with the new paint [88]. Painting is usually a part of a major renovation in buildings, which could benefit health by creating a better indoor environment with strategies of removing dust, house dust mites, molds etc.

The elderly in our study reported less pollen allergy, furry pet allergy, current rhinitis and respiratory infections in the last 3 months. The prevalence of environmental factors were also different. Two review articles have summarized articles on environment factors and respiratory health among the elderly [47], [48]. The first review summarized 59 articles about the adverse respiratory effects of outdoor air pollution among individuals >65 years [47]. The second review included 33 relevant publications about exposure to major indoor air pollutants and respiratory diseases [48]. Few studies are available on adverse effects of building dampness and molds in relation to respiratory symptoms among subjects above 60 years [48]. Building dampness (dampness index) were associated with current rhinitis, wheeze, day time breathlessness, current asthma and used antibiotic medication for respiratory infections in the last 12 months among subjects less than or equal to 65 years old in our study (born during1943–2008), but no significant association were found among subjects more than 65 years old (born before 1943). The possible explanation for our finding could be that the effects of dampness problems and renting apartments were different between elderly and others in Sweden. The elderly in our study was born before 1943, and others were born during 1943 to 1990. The major increase in atopic diseases in Sweden started among those being born in the early 1960's and later [56], [89]. The era with a lot of new chemical products started in the 1940s'. The elderly in our study was grown in a different environment (more exposed to a farming environment) compared with those younger (less than or equal to 65 years old), and which may make the elderly becoming less sensitive to building dampness and molds later in life. Sweden was a rural society in the old time and those born early were more often living on farms when they were young. Farming life experience in childhood was associated with a reduced risk of atopic sensitization [90] and less adult-onset of asthma [91].

## Conclusions

Factors in multi-family dwellings, especially rented apartments, certain building years (especially 1961–1985), building dampness and molds, window pane condensation and odor perceptions at home can be risk factors for rhinitis, asthma and respiratory infections. Preventive measures could include avoiding building dampness and molds, repairing those buildings built during the 1961–1985 and increase ventilation flow in indoor environment.
